# The right place? Users and professionals’ constructions of the place’s influence on personal recovery in community mental health services

**DOI:** 10.1186/s13033-018-0209-z

**Published:** 2018-05-31

**Authors:** Ingrid Femdal

**Affiliations:** 1 Department of Health and Social Work Studies, Østfold University College, 1671 Kråkerøy, Norway; 2 Department of Public Health and Nursing, Norwegian University of Science and Technology, 7491 Trondheim, Norway

**Keywords:** Community mental health services, Personal recovery, Place, Productive power, Social constructionism

## Abstract

**Background:**

Current mental health policy emphasizes the importance of community-based service delivery for people with mental health problems to encompass personal recovery. The aim of this study is to explore how users and professionals construct the place’s influence on personal recovery in community mental health services.

**Methods:**

This is a qualitative, interpretive study based on ten individual, semi-structured interviews with users and professionals, respectively. A discourse analysis inspired by the work of Foucault was used to analyze the interviews.

**Results:**

The findings show how place can be constructed as a potential for and as a barrier against recovery. Constructions of the aim of the services matter when choosing a place for the services. Further, constructions of user–professional relationships and flexibility are important in the constructions of an appropriate place for the services.

**Conclusions:**

The aim of the service, the user–professional relationship, and flexibility in choosing place were essential in the participants’ constructions. To find “the right place” for mental health services was constructed as context-sensitive and complex processes of assessment and co-determination.

*Trial registration* The study is approved by the Regional Committee for Medical Research Ethics, Norway (REK-Midt 2011/2057)

## Background

Current mental health policy emphasizes the importance of community-based service delivery for people with mental health problems to encompass personal recovery [1]. The place community mental health services are provided in can be understood as materiality, as a physical place. In this paper, however, place is in addition considered as social constructions. Previously, place and space have been described as emerging after hospitalization to offer shelter and care for people with mental problems [3, 4]. This study contributes to the research on place and space by focusing on constructions of how place in community mental health services impacts personal recovery by focusing on place as a social construction.

### Place and recovery in the context of community mental health services

Recovery has become a forefront of the policy agenda in many countries [1, 2], and is central to hopes for progress in mental health policy. The World Health Organization (WHO) claim that “community-based service delivery for mental health needs to encompass a recovery-based approach that puts the emphasis on supporting individuals with mental disorders and psychosocial disabilities to achieve their own aspirations and goals” [1], p. 14. Community mental health service is supposed to promote independence, belonging, and to strengthen the ability to cope with life [5], p. 7, reflecting expectations for progress. Personal recovery is described as subjective and unique, taking place within the context of the person’s everyday life [6]. It is defined as “a deeply personal, unique process of changing one’s attitudes, values, feelings, goals, skills, and/or roles. (…) a way of living a satisfying, hopeful, and contributing life even with limitations caused by illness” [7]. Davidson and Roe [8] identified two different meanings of recovery: “recovery *from”* mental illness and “recovery *in”* mental illness. The notion “recovery from” is based upon people who had been given diagnoses of severe mental illness becoming symptom free and not returning to the patient role. By contrast, the notion “recovery in” derives from the Mental Health Consumer or Survivor Movement and refers to the person’s own effort and work in getting on with life and creating a life in a community despite their mental health problems [8]. “Recovery from” can be understood in terms of medical discourses, focusing on symptoms and disease, while “recovery in” emphasizes discourses that are social and existential. From a “recovery-in” perspective, professionals are expected to use their skills and expertise in collaborative partnerships with the person’s own change process [9]. These collaborative relationships take place and are constructed in multiple ways. Cresswell [10] claims that “place is a word that seems to speak for itself” and there has been a rather shallow understanding of what the word place means. He argues that place is space invested with meaning in the context of power [10]. Place-making is seen as a process of transforming “space” (that is no-place) into “place” [11].

Gieryn [12] describes place as having three defining features. Place as a geographic location refers to a distinct physical spot (ibid.). People attach meaning to geographic locations through their own experiences and understandings, and through social, cultural and economic circumstances [13]. Secondly, place has a material form, like a living room or an office. Social processes, such as power, happen in the material forms we design, build and use [12]. The third feature of place is investment with meaning and value. Gieryn [12] claims that without naming, identification or representation by people, a place is not a place. Place is described as an investment with meaning, and value appears in constructions of place as lived experience, sense of belonging, social place and healing place. Place as lived experience, implies that people experience it in distinct ways, like when one person experiences a place different at different times, as the experiences and constructions of place are changing [10, 13]. Place as ‘the meaning and significance people accord to a specific place’ refers to a sense of belonging [14], p. 204. Constructions of place sometimes demonstrate the meaning of relationships, like place as a healing place. As such, it is constructed as a social place in terms of interpersonal relationships involving user and provider [14]. The nurse’s presence is considered as a therapeutic enterprise and as contributing a unique and comforting element to the person’s illness experience [14, 15]. Constructed as a social place, place refers to interpersonal relationships developing between user and provider, and between relatives and provider. Such relationships are central in the current discourse on mental health services [16, 17]. In this discourse, the ideal relationship is presented as supporting personal recovery [18].

Community based mental health services are supposed to encompass a recovery-based approach, based on the individual’s own aspirations and goals [1]. One of the places to do so is in the user’s home. The home environment represents a multitude of meanings, like personal security, privacy [13, 18–22], comfort, control [19], identity, [22] and autonomy [20, 21]. The notion home is not just spaces in a mundane sense. Rather, it is imbued with emotions, relations and histories [23]. Home as lived experience, space and social place means that the constructions depends on the power relationships between the involved persons. Professionals’ constructions of a user’s home may be based on what the user’s home look like, if it is neat and clean or messy and unclean, how it is decorated with items, furnishings and other effects. A home visit is thus a professional tool to enter private home-spaces, observe and collect data required for professional assessments [4]. Physical structures may accordingly be seen as representing underlying social structures and other invisible divisions [13]. Dyck et al. [19], p. 175 claim that “the home’s materiality is also a signifier of a person’s location within power relations that influence access to material resources, as well as culturally valued consumer goods”. Healthcare delivery in a person’s home is frequently cast as an invasion of privacy or an intrusion on a person’s space [24]. Some claim that the privacy of the home is challenged and, as a consequence, the user’s ability to restrict public surveillance is compromised [13]. However, professionals’ sense of power, authority, control and sense of a controlled workspace may also be altered [13].

Mental health service can be provided in mental health workers’ offices and other places in addition to the users’ homes [25–27].

Some perceive the professional’s office as a formal place [25] and emphasize the potential of using the users’ surroundings in an effort to achieve good contact or practicing on everyday situations the users perceive as difficult [27]. Some use public places such as shops and cafés to practice the management of situations the users find extremely discomforting, like going out among other people [26].

## Care relationships and productive power

“Care and care relationships are located in, shaped by, and shape particular spaces and places… (..)”, according to Milligan and Wiles [28], p. 736. To develop an understanding of how relationships and power shape the constructions of places’ meaning for individual support, the work of Foucault [29, 30] is applied. Foucault was interested in power as an omnipresent and relational phenomenon and studied power at the level of society as a whole as well at what he referred to as “the capillary level of power” [29]. He claimed that power is always present in human relationships and preferred the phrase “***relations of power***” over “power” [30]. Power relations are described as mobile, reversible, and unstable [31]. Thus, power is unavoidably present in all social relations, such as those between users and professionals, and users’ and professionals’ actions and beliefs shape and are shaped by such power relations. “We cannot jump outside the situation, and there is no point where you are free from all power relations. But you can always change it” [30].

In mental health services power often has negative connotations, and is associated with restricting other people’s freedom, domination, control and coercion, in a hierarchic system where health professionals possess power [32]. According to Foucault however, power is not a substance or a property one can claim to possess. Within such a perspective, power is not understood as repressive, but rather as a productive force that promote actions [33]. However, in this paper I draw on Foucault’s notion of power as “to be able to”. In English, the term power refers to both the capacity to do something and to the act itself [34]. The French language, by contrast, has two terms to decipher power, puissance and pouvoir (…), where broadly the former denotes capacity and the latter denotes the act of power [34], p. 105–106). Elden [35] claims that Foucault attempts to capture the creative, productive sense of power by using the French word pouvoir meaning, “to be able to”.

### Aim

The aim of this paper is to explore *how users and professionals construct the place’s influence on personal recovery in community mental health services*. It follows from this that the intention is not to present the true right place for these services, but to present how “the right place” is co-constructed by people involved in community-based community mental health services.

## Methods

This qualitative, interpretive work using individual, semi-structured interviews was designed to explore how the place influence personal recovery in community mental health services from the perspectives of both users and professionals.

### Recruitment of participants and sample

Participants were recruited through the public community mental health services at municipality level, based on the acceptance of the service leaders. To meet the inclusion criteria, professional participants had a bachelor’s degree in a health or social profession and work half- to full-time position for at least 6 months. The service leaders recruited mental health workers who met the criteria. Inclusion criteria for service users were adults (> 18 years), assisted by community mental health services at least once every other week for at least 2 months, diagnosed with schizophrenia, psychosis or moderate/severe depression, who would give informed consent. The users’ contact persons at the community mental health care service recruited users to the study. The researcher received contact information only to users and professionals when they accepted to participate in the study. The material presented here is based on 10 users and 10 professionals, seven women and three men in each group.

### The interviews

The participants chose the place for their interview: in the participant’s home, at the community mental health care center or at the interviewer’s workplace. The author, who is a mental health nurse and a Ph.D. candidate, did the interviews. The themes in the interview guide focused on experiences of user–professional interaction and cooperation to promote reflections on roles, expectations, opportunities and experiences. Questions were asked from the themes in the interview guide. The interviews lasted for 45–60 min, and were audiotaped and transcribed verbatim by the author.

### Data analysis

A discourse analysis inspired by the work of Foucault was used to analyze the interviews. By using the six stages developed by Willig [36] the author performed the analysis manually. Initially, the text was read without any purpose of analyzing. To understand the constructions of places’ influence on personal recovery in community mental health service was the discursive object and a basic point for the analysis. The aim in stage 1 was to identify all the ways the phenomena of study were described and constructed. I explored how the discursive objects were constructed through language, and what types of objects that were constructed. In stage 2, various discourses behind users’ and health professionals’ constructions were identified by focusing on the differences between their constructions and by asking what discourses the constructions were based on and what their relationship to each other were. In the third stage, I analyzed the achievements of the constructions and what is gained by deploying them in this position. In stage 4, I asked why the participants positioned themselves as they did in the situations to understand how place and actions opened up or closed down opportunities for action. Stage 5 maps the opportunities provided by this positioning. In the sixth stage, I wanted to identify the subjectivity of the object when placed in the specific positions. The focus here was to detect what potentially can be felt, thought and experienced from the available subject positions [36]. The author used the input of colleagues to temper personal biases. The steps are shown in Table 1.

**Table 1:** The analysis process, based on Willig’s six-stage analysis model

Stage 1	Discursive constructions	How were the place’s influence on personal recovery constructed in the data?
Stage 2	Discourses	What discourses are the statements drawn upon and how are they related?
Stage 3	Action orientation	What do the constructions achieve and what is gained from deploying them?
Stage 4	Positioning	What subject positions are made available by these constructions?
Stage 5	Practice	What possibilities for action are mapped out by these constructions?
Stage 6	Subjectivity	What might the users and professionals feel, think and experience from the available positions?

### Ethical considerations

The guidelines of the Helsinki Declaration have been followed and the study is approved by the Regional Committee for Medical Research Ethics (REK-Midt 2011/2057). Ethical approval of the project was given with the requirement that users should be recruited by professionals who were not themselves involved as participants in this study. All participants gave their written consent prior to the interviews and they were informed about the opportunity to withdraw from the study at any time without consequences. Participants’ names in the findings are fictional.

## Findings

The overall impression working with the data is that the constructions were related to the place’s meaning and potential for personal recovery and not on the physical place as such. By studying the dynamics of place and recovery, processes of user–professional relationships came to the fore. The findings reveal two main areas when the participants constructed the place’s influence on personal recovery in community mental health service: the place as a potential for recovery and as barriers against recovery.

### Constructions of place as a potential for recovery

User–professional relationships were often involved in the participants’ constructions of place as a potential for recovery. The meaning of the relationship comes to the forefront, when Hanna (user) explains how she feels when Caroline (professional) visits her at home:

“I don’t have to be stressed doing housework before Caroline comes. You want to make a good impression when you are having guests. It is not like that when she comes. You don’t have to make up an excuse and delay the visit—just because you could not handle the housework. She is not a friend. It is something else” (Hanna, user).

By her statement, Hanna demonstrated that she did not feel pressure from too great expectations when the professional visit her at home. Rather, she felt more relaxed than when she is having guests. She created a position for the professional that released herself from duties expected to be fulfilled when having guests.

The user’s autonomy and right to decide is essential in the participants’ constructions of the place’s influence on personal recovery when professionals visits users at home. Professionals cannot take it for granted to be invited in, Hanna (user) claims; “It is up to me to unlock the door.” (laughs). “Or else they cannot come in. When you are feeling down, it is easy to lock the door and close the curtains”. By this statement, Hanna showed that she is in charge to decide whether she wanted to give the professional access to her home or not. Furthermore, the participants describe a transformation of users’ and professionals’ roles and positions when a user receives services in his or her home.

“A user can say; “It’s enough”, and I have to comply to his or her wishes. In my opinion, they have more power at home than here (…). “This is my home. I smoke whenever I want to”. In fact, that is difficult. To be in someone’s home when he or she smokes a lot. To have to change clothes after you have left. Their autonomy… where you can sit, where to go… it is their homes. They decide. When you are there, they control you a little as well. I have my space, but it is far less than the user’s. This is my space. I have to ask for permission. “May I use the bathroom?” I cannot just take another piece of cake or coffee if the user has not asked me to” (Lars, professional).

By asking for permission and expecting the user’s rules to be dominant, Lars positioned himself as a visitor in the user’s home. In a home, there is no public smoking law. The resident decides—it is his or her domain. These constructions differs from a traditional paternalistic one, where professionals’ make the rules.

The aim of the service needed was important in the constructions of the place as a potential for recovery. The participants constructed places as motivating and empowering for the users. Some described the importance of having an appointment at the community mental health center as a way to become motivated.

“When I have an appointment, it helps me to get out. Get up in the morning, get out of the house, and make sure I get there on time. When I am ill and feeling down, I usually stay at home. It is of great importance, especially in situations like that, to get out”. (Greta, user)

The way to or back from the mental health care center was sometimes described as an opportunity to practice skills they struggle with, like doing some shopping on the way home, taking the bus by themselves or talking to someone they meet on the way. Finding solutions together and to be flexible in order to meet the users’ challenges in their everyday lives were described as motivating for the users. “When a person feels that it is too hard to come, I visit them at home” (Emma, professional). Lars (professional) claimed: “If they don’t manage to come here themselves, I sometimes ask if they want me go along with them… if they don’t have someone to accompany them”. By this quotation, the journey from the user’s home to the community mental health care center is constructed as a way to overcome an obstacle.

Even measures that are usually negatively connoted, as being controlled in their own homes, could be motivational for some of the users:

“They came to check if I have done the things we agreed on. (…) When it happened, it was all right. I had an excuse to clean and clear up. It would be difficult to do it if they did not come to check. It is a kind of motivation”. (Anna, user)

Professionals expected themselves to motivate users to keep up with their housework, and looked for solutions that could motivate and empower the users:

“Sometimes I suggest that we do the dishes as we talk about what the week was like, when it is messy in a person’s home. It is part of working on the relationship, doing something practical and helping the person doing some housework” (Linda, professional).

This statement presents doing housework together as serving several purposes: It can be a way to reflect on the passing week, work on the relationship, and to clean up the house. The disorganized home can be interpreted as a sign of mental illness, and doing homework together as attempt to make the user active and participating again. However, the professional do not do the housework on behalf of the user but expect him to participate, which can be interpreted as part of a user participation discourse.

The participants did not only construct the user’s home or an office as places with potential for recovery in mental health services. Participants explained that meeting in other places, like sitting on a bench outside the user’s home, in the woods, go for a walk or at a café, could be a way to create a safe environment in which they could be exposed to the things the user fear or avoid. The professionals positioned themselves as the ones making the suggestions, even though they rather wanted the users to do so themselves; “Usually, the idea comes from me. Nevertheless, sometimes they make proposals after a while. Some have suggestions as to where they would like to go. I encourage them to do so. Usually, they are my suggestions” (Erik, professional). By being the one who makes the suggestions, Erik takes the position as the one who govern the process. He tries to encourage the user to make the same suggestions as he did as well.

Professionals argued that it was necessary to visit a user at home in order to get important information about a person’s everyday life with mental health problems in some situations.

“When a person is at home, it is easier to understand what he or she struggles with. They can sit in my office saying everything is all right at home, or just the opposite. When you come home to a person, you make an assessment yourself” (Lars, professional).

The statement may be interpreted as a sign of distrust in the user, as the professional did not trust the user to come forward with the important information themselves. At the same time, it can be understood as a way to learn to know the user and the place’s influence on how the user cope with their mental health problems. Many of the users lived in families. The presence of family members at home was used as an argument to make the user come to the professional’s office: “Often the users want it themselves. ‘My wife is at home, too. I would rather not talk about my problems when she is present.’ So we talk about his or her situation and find a solution” (Marie, professional). In this case, the user is an active part of the decision-making. “At home, the telephone is calling, there are things they need to do. The concentration on the conversation is much better here. Here we have provided a place and time for the conversation” (Hilda, professional). By this argumentation, Hilda underline the importance of being flexible and cooperative without interruptions.

### Constructions of place as a barrier against recovery

As in constructions of place as a potential for recovery, user–professional relationships were often involved in the participants’ constructions of place as a barrier against recovery. The Place was described as somewhere the participants felt vulnerable or uncomfortable. “First, you have to open up your home, and then you have to open up yourself. I think I would have felt more vulnerable if she came to my house” (Siv, user). By talking about her feeling of vulnerability if the professionals came to her house, an asymmetry in the relationship appears. The professionals did not open their homes for the users. She said she feels extra vulnerable and nervous when the professional came to her house.

“There was a time I did not make myself dinner. It was not because I did not know how to. It was because I would rather not eat. I remember a health worker who came to my house to help me make dinner. They said I needed to practice cooking. I did not want it to happen, but I did not dear to say no. Wanted to be a good client. Was afraid of the consequences if I resisted… I was afraid of several things. I was afraid they would be mad at me, that it would lead to many negative things. That Tobias (fictive name on the professional) would be mad and talk about it a lot. Even worse… Maybe he did not want to talk to me again” (Siv, user).

Siv talked as if the professionals took for granted that they knew what was best for her—even without asking about her opinion. The fear for the consequences seemed to surpass the fact that she did not want the professionals to come to her house to practice cooking. By putting pressure on Siv and not ask for her opinion, they demonstrated a rather paternalistic attitude.

Some professionals offered the users services at their office to save time. By doing this, they had time for more users. This can be interpreted, as the need to save time and be effective was superior to the users’ individual needs. In accordance, some professionals claimed they were concerned about how to end a user–professional relationship to avoid that the number of users accumulates. This way, the users’ individual needs become subordinate to the system and the need to save money. At the same time, a dominant understanding among users and professionals was that it takes time to get to know each other well and to establish a good alliance. A good user–professional relationship based on trust was described as a prerequisite to feeling safe and comfortable, regardless of the physical place.

It was a dominant understanding by users and professionals that users felt vulnerable and uncomfortable in some situations and at some places. However, the professionals were also constructed as vulnerable and scared:

“Health workers can feel threatened and be afraid of accusations when they visit a person at home. Sometimes we go two mental health workers when we feel insecure… Sometimes sexual matters affects the situation. You can be accused of things you have not done. I have never been in a situation like that, but sometimes I am afraid to be accused of having other intentions than I have. When it comes to avoiding physical attacks, it is smart to be two, too”. (Martin, professional)

In this quote, Martin talks about ways to protect himself from false accusations and physical attacks. At the same time, he position users as dangerous and unpredictable. What he does not talk about is the user’s reaction to this behavior, like when two professionals visit them in their own homes. This behavior may reinforce the differences, as the user does not necessarily have support from a partner. Accordingly, the professional’s fear and insecurity can work as a barrier against the user’s recovery. The procedure at the professionals’ offices at mental health centers could also reinforce the differences between user and professional:

“Usually they come to talk in my office. (…) I go to the waiting room to bring him or her to my office when it is time, because the doors are locked. I show him or her to my office and the conversation takes place here. I use to sit here and the user there (points at the chairs”) (Anna, professional).

Differences in the roles and the surroundings can be seen as reflecting differences in power: One is coming to get help; the other is supposed to help. “The person who needs help is at the provider’s office”, The mental health worker is in possession of an office. He or she waited for the helper to unlock the door; the helper has a key to unlock the door. One decides where to sit; the other person was offered a place to sit. The asymmetry is even more obvious when she later tells that the staff and the people seeking their help use separate toilets: “Yes, of course. The public toilet is down the hall. Ours is right over there”. In these discourses, Anna talks about the asymmetry as self-evident. The statement underlines the differences between helpers and people coming for help, it becomes a “they” and “us”. The distance became evident through the asymmetry described by where they meet and what the room is like:

“It depends on the setting… This is kind of my domain. This is my office. We also have a meeting room. I don’t like it so much because it is cold and impersonal. In our office, there are chairs and a little table in the middle. It is so much more comfortable… I don’t like to talk with clients the way we sit right now (in the meeting room, chairs on each side of the table). It is almost like an examination. And I don’t mean to examine them” (Lars, professional).

By this statement, Lars suggests that any room at the center is appropriate, regardless of the design and functions of the room. He shows how the room can work as an obstacle to recovery if the way they sit and talk feels like an examination.

## Discussion

In this study, places were created as frames the person recovers in. The constructions of place were not necessarily located to a specific geographic location, rather to the function the place had in a person’s recovery process. Constructions of place can be considered as fluid and relational, with experience and actions understood as produced distinct from rather than within space [23].

The study demonstrates that users and professionals constructed place as a social place in terms of interpersonal relationship between user and professional. The importance of creating and preserving a good, trusting user–professional relationship is underlined in the literature [4], as it is in this study as well. The findings illuminate how the choice of place sometimes is taken for granted when the professionals assume they know what is best for the users to recover in. They are told, rather than provided an opportunity to engage in a relationship that is reciprocal, to influence on their recovery process. Perkins and Slade [37] claim that a good relationship is very difficult when power is not acknowledged or addressed through the process. To be treated as a person and not as an object gives the patient a feeling of being respected [20]. However, the professionals act in different ways to govern users; to make and keep users active, participating, enterprising and self-governing, and users respond and take part within the same discursive framework [38]. Powers [39] argues that empowerment improves the ability of people to be governed. She claims that despite the ideas of redistribution of power are emphasised in contemporary health care, power relations are not changed, and practices of empowerment end in dependency (ibid.). A user–professional collaborative treatment process towards the user’s expectations for treatment and personal aims for treatment and life, is claimed to be important to recover from mental health problems [26, 40].

In the study constructions of user–professional relationship was essential when a place was chosen, showing that the power relation can open up or close the possibilities to develop, regardless of the physical location. By choosing place related to the goals for the interaction, the users were be able to work on challenging situations. This is an example of how power is productive and not just repressive, in line with Foucault’s notion of power as strategic acts that encompass all directions [29]. Community nursing means thinking about how place matters in the users’ lives and ask them about the meaning that particular place hold for them [13].

The study illuminates how the constructions of the user’s home as the place for user–professional interactions lead to diametrically different directions: as safe and comfortable or as surveillance and a stressful situation. Usually, a home is considered as a private place, a place from which they can exclude unwanted outsiders [13]. There may be a risk that routines and security requirements that originate from hospital norms and values will be transferred to the home without critical reflection [41, 42]. What was meant to be a part of deinstitutionalization might be considered as an institutionalization of a person’s home, and the home space become more “public” [19]. There has been warned against modelling home care for people with mental health problems on institutional care [43, 44]. Heritage [45] claim that professionals perform their institutional identities in the users’ homes, when they use topics during visits which have an institutional agenda (like goal orientation, targeted and based on care plans), or when they follow certain procedures discussing and checking the condition of the clients and their apartments. As a consequence, users can find it degrading when they have to adapt their activities in their own homes to the routines of the professionals [20]. On the contrary, social call talks without any professional aims may contribute to a “recovery in” inclusion process [4].

In this study, professionals struggle to find ways to cooperate with the users and exercise their role as professionals, leading to frustration regarding how to exercise their work as a professional, as well as paternalism. Some claim that professionals take their power for granted in user–professional relationships [13]. Juhila et al. [4], p. 106 argues that homes “can be institutionalized, if workers take a leading, authoritative, controlling and interventionist role in home encounters.” The constructions of the boundaries between public and private become blurred. On the contrary, the blurred boundaries between personal and professional in community mental health services can create spaces that engender a more egalitarian partnership between users and professionals [46]. The study also shows that professionals are challenged when there service users and professionals have competing needs. For instance, when a professional visits a user who smokes a lot in his or her home, and the professional changes his clothes before meeting the next user. On one hand, users have the right to smoke in their own homes. Decisional autonomy is believed to be one of the basic principles of an ethical health care system [47]. On the other hand, the home is also mental health workers’ work environment. The health workers are exposed to the cigarette smoke whether they want it or not. Furthermore, the smell of smoke from the health workers’ hair and clothes may feel uncomfortable to other users.

The study shows that institutional thinking has been transferred to community mental health care when professionals come to pick up the user at the waiting room, unlock the doors, lead the conversation against goals, and care plans. One can ask if the users really are able to cooperate and make autonomous decisions if the professionals set the rules, and if professionals only ‘pretend’ co-determination directed at practicalities rather than involving users in fundamental decisions [48]. This is considered as tokenistic involvement when people are led to believe that their influence is greater than it actually is.

## Strengths and limitations of the study

The strengths in this study lies in its focus on both users’ and professionals’ constructions of the place’s influence on personal recovery in community mental health services. The purpose of a discourse interview would not be to look for a true and external reality that science to a greater or lesser degree can correspond to [49]. Nevertheless, awareness of the meaning of place is important to increase reflexive and improved understandings of how place influences recovery processes.

The fact that I understand my inquiry to be inextricably bound to my positions in social worlds and in the Norwegian context in particular may be considered as a limitation of the study. For instance, most community mental health services are public in Norway. These constructions may still be transferable to others settings in other countries. The focus of discourse analysis is not to find an underlying truth, but to show how discourses operate in order to make some statements accepted as meaningful and true [36]. Truth is understood as a discursive construction and different knowledge regimes state what is true or false.

## Conclusion

The constructions show that there is no such place as “the right one” in community mental health services. On the contrary, to find places that is suitable to recovery in mental health problems is a complex process of assessments, flexibility and co-determinations. By clearing the discourses at play within the place’s influence on personal recovery in community mental health services, the existing practices in the field can be opened up to further reflections and awareness when choosing places in community mental health services.
